# The Effect of Marijuana on the Incidence and Evolution of Male Infertility: A Systematic Review

**DOI:** 10.7759/cureus.20119

**Published:** 2021-12-02

**Authors:** Mirra Srinivasan, Ranim K Hamouda, Baba Ambedkar, Hadia I Arzoun, Isra Sahib, Jack Fondeur, Lisbeth Escudero Mendez, Lubna Mohammed

**Affiliations:** 1 Internal Medicine, California Institute of Behavioral Neurosciences & Psychology, Fairfield, USA; 2 Pathology, California Institute of Behavioral Neurosciences & Psychology, Fairfield, USA

**Keywords:** endocannabinoid system, hypogonadism, semen analysis, male reproductive health, male infertility, marijuana

## Abstract

Over the past decade, the consumption of marijuana or any other form of cannabis, whether medically, recreationally, or illegally, has been escalating worldwide. The additive effect of marijuana and the easy availability could make this increasing trend possible for imperceptible outcomes affecting one's physiology on multiple levels. The rationale of this review is to study and enumerate several effects marijuana may have on male reproductive organs, especially in men who are dealing with subfertility or infertility issues. A literature search was done from September 1, 2021, to September 14, 2021, using the following databases: PubMed, Google Scholar, Bielefeld Academic Search Engine (BASE), University of California, Santa Barbara Library, and PubMed Central. The studies included in this review comprised systematic reviews, cross-sectional, case-control, cohort, and longitudinal studies published during 2010-2021 in the English language. After an extensive review of all studies, the quality was assessed using appropriate quality appraisal tools, and 15 eligible reports were identified and included.

In-depth research on the final studies concluded that marijuana seems to have specific adverse effects on the sperm parameters, namely, sperm count, concentration, motility, morphology, capacitation, and viability, thus affecting fertility in men. Certain hormone levels, including testosterone, luteinizing hormone, and follicle-stimulating hormone, also drew attention, potentially impacting men's fertility; however, a finite inference could not be substantiated by the studies. Although the studies show significant effects in sperm parameters and organic sexual dysfunction, it is also to be noted that these studies are observational only and are conducted in small groups in multicenter geographical locations where other lifestyle patterns could be confounding. Given this restriction, it is suggested that further human trials on a larger scale be conducted to provide an even more concrete conclusion, especially after considering other factors that may affect the generalization of these trials.

## Introduction and background

"Cannabis has been split, in the public imagination and in many of our laws, into a good guy and a bad guy, a drug and a medicine." - Amber Senter [1].

Cannabis, also known as marijuana, is the most extensively grown, traded, and abused illicit substance in the United States and worldwide [2]. In the last decade, cannabis misuse has increased at a higher rate than cocaine and opiate abuse. About 147 million people, or 2.5% of the world's population (annual prevalence), consume it compared to 0.2% who use cocaine and 0.2% who use opiates [3]. In the United States, 48.2 million people (about 18%) were consuming marijuana at least once in 2019 [4].

The dried flowers, leaves, stems, and seeds of the cannabis plant are also known as marijuana, pot, or dope [5]. The principal psychoactive ingredient in marijuana is delta-9-tetrahydrocannabinol (THC), responsible for most of the intoxication effects that individuals seek. More than 500 substances are found in the plant, including more than 100 cannabinoids (CBs) chemically related to THC [6]. Next to alcohol and tobacco, marijuana has been a highly addictive drug that stimulates the psychoactive behavior of an individual. According to the recent research, 1 in 10 marijuana users will become addicted, and if 18 years or younger, that changes to 1 in 6 [7-9]. This number is on the verge of an increase, and with limited restrictions on the availability of this substance, it may even bring about counterproductive effects that could be unknown.

**Figure 1:**
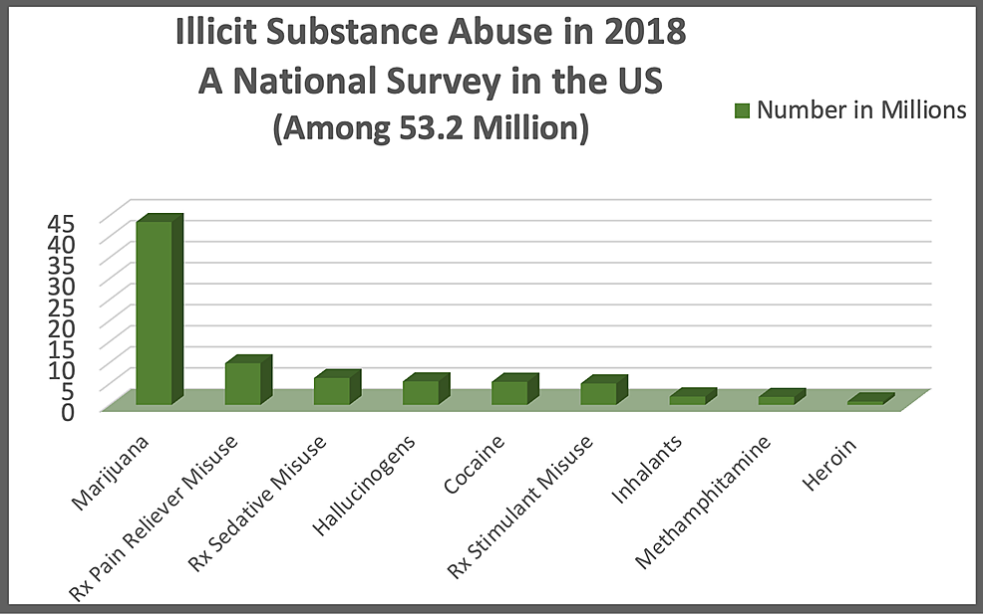
Illicit substance abuse in 2018 Source: Key substance use and mental health indicators in the United States: results from the 2018 National Survey on Drug Use and Health [10]. The estimated numbers of various illicit drugs are not mutually exclusive, as people may have used more than one sort of drug.

Figure 1 depicts the various illicit drugs used in the United States in 2018 [10].

**Figure 2:**
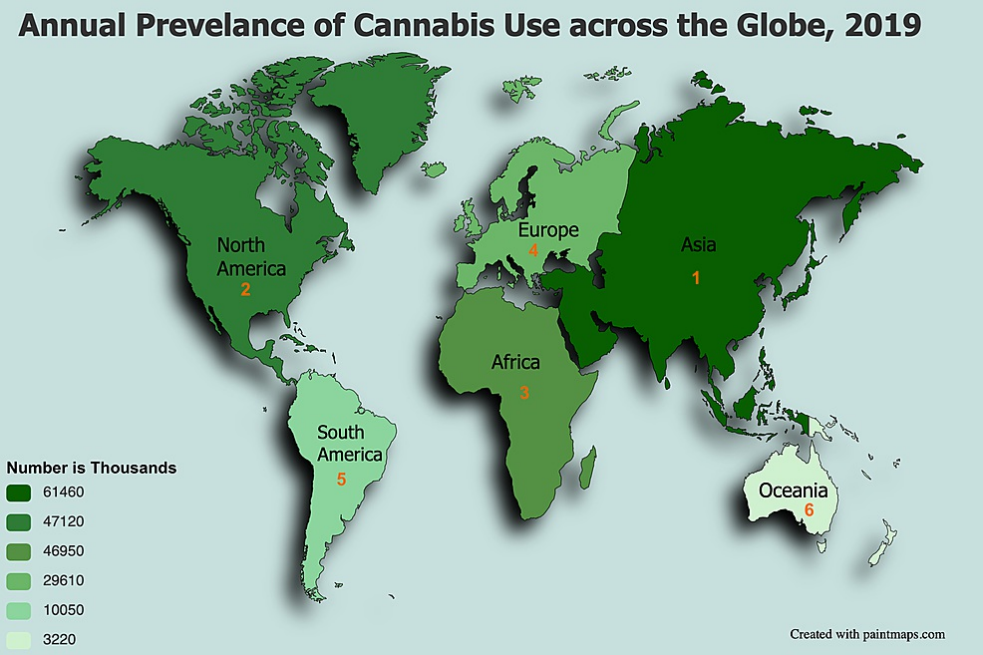
Annual prevalence of cannabis use across the globe in 2019 Source: World Drug Report 2021 [11].

Figure 2 depicts the annual prevalence of cannabis use across the globe in 2019, based on annual report questionnaire data and other official sources [11].

**Figure 3:**
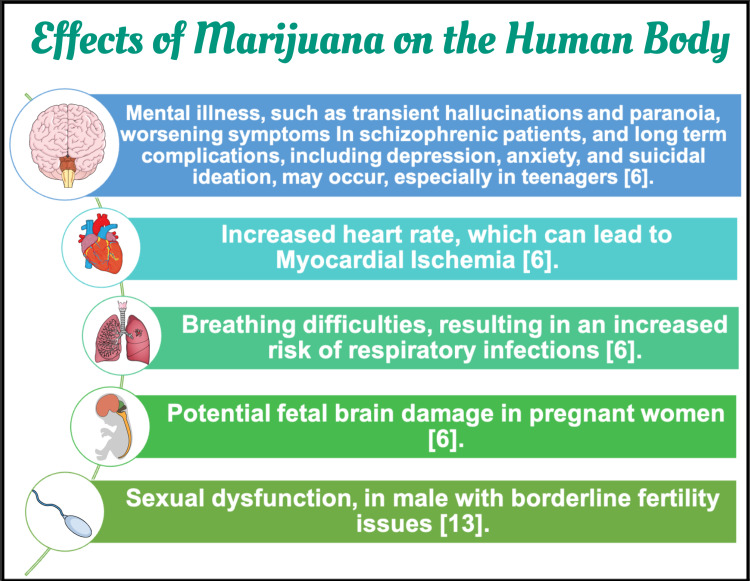
Effects of marijuana on the human body Image created in Mind the Graph platform.

The world has been evolving to many discoveries in the health care field where marijuana has been medically prescribed in disorders such as pain, spasticity associated with multiple sclerosis, nausea, posttraumatic stress disorder, cancer, epilepsy, cachexia, glaucoma, human immunodeficiency virus (HIV)/acquired immunodeficiency syndrome (AIDS), and degenerative neurological conditions. However, aspects of this active substance have to be studied and further understood [12]. Studies show that marijuana usage can be linked to several adverse health consequences in a multi-system pattern [6,13], as illustrated in Figure 3.

Infertility affects millions of people in the reproductive age around the world, distressing families and communities. According to estimates, infertility affects 48 million couples and 186 million people worldwide, with a male factor accounting for roughly half of the instances [14]. Male infertility can be caused by many factors that interfere with spermatogenesis, organic dysfunction, and psychogenic causes, including inherited, acquired, and idiopathic reasons [14]. All of these factors, as well as age, drugs, surgical history, exposure to environmental contaminants, genetic abnormalities, and systemic illnesses, can impact reproductiveness.

In today's fast-paced lifestyle, people often fail to think about the long-term effects of their actions. The younger generation may not realize it at an adolescent age, but these effects may be unfavorable for most individuals. One such repercussion can be male infertility with marijuana use. The cardinal ingredient in marijuana, tetrahydrocannabinol, a lipophilic molecule, interacts with the cannabinoid receptors in the endogenous endocannabinoid system (ECS), found in the hypothalamus, pituitary, and internal reproductive organs of both females and males [15]. The presence of these cannabinoid receptors on sperm shows cannabis has the potential to not only disrupt sperm function but also alter reproductive hormones, semen characteristics and decrease desire and sexual performance. This decreased libido may impact mental health leading to more relationship problems, increased stress, and poorer physical health, which may, in turn, cause sexual dysfunction from a psychological viewpoint, apart from affecting the semen quality [16].

This review article analyses the possible adverse effects of marijuana on male infertility, with a particular focus on whether cannabis use affects the quality, motility, volume, and survivability of sperm and alterations in hormone levels.

Methodology

This systematic review follows the Preferred Reporting Items for Systematic Reviews and Meta-Analyses (PRISMA) guidelines and principles [17].

Inclusion and Exclusion Criteria

The choice of studies included were systematic reviews, cross-sectional, case-control, cohort, and longitudinal studies published during 2010-2021 in the English language only. The population group comprised males in the reproductive age group, and the population, intervention, comparison, outcome, and study criteria (PICOS) were incorporated. Non-English, non-full text articles, animal studies, female factors contributing to infertility, and confounding bias studies were excluded.

Information Sources and Search Strategy

This study explored five databases: PubMed, Google Scholar, PubMed Central (PMC), Bielefeld Academic Search Engine (BASE), and University of California, Santa Barbara Library. After an in-depth exploration, all the relevant articles were procured electronically using appropriate keywords, including the medical subject headings (MeSH) strategy. The Boolean scheme was used to vitalize the keywords, and the MeSH strategy format was applied in PubMed. The retrieved articles were thoroughly checked for the titles, abstracts, subject headings, and references. Through this process, all irrelevant articles were avoided, and all pertinent reports were utilized.

Keywords

The MeSH keywords in PubMed included the following: Marijuana OR Cannabis OR (("Cannabis"[Mesh] OR "Marijuana Use"[Mesh]) OR ("Cannabis/adverse effects"[Mesh] OR "Cannabis/drug effects"[Mesh])) AND Male Infertility OR Male Hypogonadism OR ("Infertility, Male/analysis"[Mesh] OR "Infertility, Male/etiology"[Mesh]).

The keywords on other databases included the following: marijuana, cannabis, male infertility, sperm motility, fertility investigations, recreational marijuana use, sperm parameters, lifestyle, risk factors, semen quality, male reproductive health, semen analysis, spermatogenesis, hypogonadism, and endocannabinoid system.

Data Extraction and Selection Process

The study team used appropriate quality appraisal tools during the selection process to assess whether the studies met the inclusion criteria. Two researchers autonomously carried out the data selection and data extraction. This process was done independently of each other. Data collection and extraction were done from September 1, 2021, to September 14, 2021. In cases of disagreements, both researchers conferred about the study designs, inclusion and exclusion criteria, intervention used, and outcome measured to reach an agreement. In indecisive circumstances, a third reviewer was consulted to reconcile differences and reach common ground.

Quality Assessment

The studies were assessed for quality, and reports were selected with the following scores: Newcastle-Ottawa tool (cross-sectional, longitudinal, case-control, and cohort) with a score of 10 or more, PRISMA tool (systematic reviews) with a score of 22 or more, and Scale for the Assessment of Narrative Review Articles (SANRA) checklist (traditional reviews) with a score of 9 or more. Table 1 summarizes the studies assessed.

**Table 1:** Quality assessment based on appropriate appraisal tools PRISMA, Preferred Reporting Items for Systematic Reviews and Meta-Analyses; SANRA, Scale for the Assessment of Narrative Review Articles

Type of study	Tools used	No. of studies
Systematic review	PRISMA	4
Case-control study	Newcastle-Ottawa	2
Cross-sectional study	Newcastle-Ottawa	4
Cohort	Newcastle-Ottawa	2
Longitudinal study	Newcastle-Ottawa	1
Traditional review	SANRA checklist	2

Results

**Figure 4:**
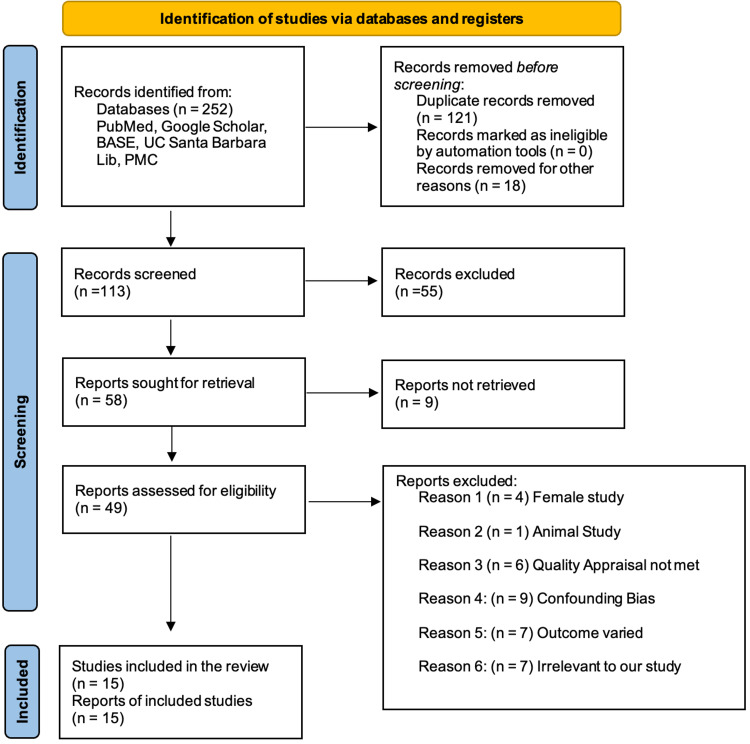
PRISMA 2020 flow diagram for new systematic reviews, which included searches of databases and registers only PRISMA, Preferred Reporting Items for Systematic Reviews and Meta-Analyses; BASE, Bielefeld Academic Search Engine; UC Santa Barbara Lib, University of California, Santa Barbara Library; PMC, PubMed Central

The search strategy used in this study, as mentioned above, included five different databases that yielded 252 articles, out of which 121 were duplicates and were removed using EndNote, 18 were removed due to ineligible records, and no automation tools were used. A total of 113 records were screened, out of which 55 were excluded based on the relevance and the inclusion/exclusion criteria. Nine reports were not retrievable, and the final screening was down to 49 reports, which were checked for quality and eligibility. After a thorough reading, 15 studies were included in the review. Figure 4 illustrates the PRISMA flow diagram and the search process used in this study [17].

Table 2 summarizes the findings and conclusions from each of the studies examined.

**Table 2:** Summary of study findings ECS, endocannabinoid system; FSH, follicle-stimulating hormone; ART, assisted reproductive technology; ED, erectile dysfunction

Author	Year	Title	Method	Subjects	Findings/conclusion
Ugboma et al. [18]	2012	Adolescent cannabis use–a young adult and middle age urologic and reproductive dilemma: the Niger delta malady	Case-control study	2290	Cannabis use was associated with urological dysfunctions like ED, infertility, and testicular cancer.
Lewis et al. [19]	2012	Differences in the endocannabinoid system of sperm from fertile and infertile men	Cohort study	150	Fertility outcome was affected due to unprecedented alterations of the ECS in infertile sperm, impacting the capacitation and acrosome reaction.
Pacey et al. [20]	2014	Modifiable and non-modifiable risk factors for poor sperm morphology	Case-control study	318	The study found few modifiable factors linked to poor sperm morphology. The only practical suggestion for men trying to conceive is to minimize their cannabis exposure if they are chronic users.
Alvarez [21]	2015	Do some addictions interfere with fertility?	Traditional review	NA	Detecting and correcting unfavorable lifestyles and toxic factors is fundamental for improving spontaneous fertility and ART results.
du Plessis et al. [22]	2015	Marijuana, phytocannabinoids, the endocannabinoid system, and male fertility	Traditional review	NA	Marijuana and its compounds can influence male fertility at multiple levels.
Gundersen et al. [23]	2015	Association between use of marijuana and male reproductive hormones and semen quality: a study among 1,215 healthy young men	Cross-sectional study	1215	Marijuana use may contribute to the etiology of the recently reported high frequency of subnormal human sperm counts.
Thistle et al. [24]	2017	Marijuana use and serum testosterone concentrations among U.S. males	Cross-sectional study	1577	The findings show that the link between marijuana use and serum testosterone is greater in men aged 18–29 years. This age group has an increased risk of nonseminoma cancer.
Rajanahally et al. [25]	2019	The relationship between cannabis and male infertility, sexual health, and neoplasm: a systematic review	Systematic review	NA	Cannabinoids seem to play an inhibitory role in regard to male factor fertility.
Nassan et al. [26]	2019	Marijuana smoking and markers of testicular function among men from a fertility center	Longitudinal study	662	Marijuana smokers had higher sperm concentration and sperm count, lower prevalence of sperm parameters, and lower FSH concentrations than those who were never marijuana smokers.
Carroll et al. [13]	2019	Marijuana use and its influence on sperm morphology and motility: identified risk for fertility among Jamaican men	Cross-sectional study	229	Recent use of marijuana as well as moderate to large quantities had an impact on sperm motility and morphology in men being investigated for infertility.
Pizzol et al. [27]	2019	Relationship between cannabis use and ED: a systematic review and meta-analysis	Systematic review	NA	Data suggest that ED is twice as high in cannabis users compared to controls.
Payne et al. [16]	2019	Cannabis and male fertility: a systematic review	Systematic review	NA	Cannabis seems to have a negative impact on male fertility.
Belladelli et al. [28]	2020	The association between cannabis use and testicular function in men: a systematic review and meta-analysis	Systematic review	NA	The study suggests a negligible clinical effect of cannabis use on testicular function.
Hehemann et al. [29]	2021	Evaluation of the impact of marijuana use on semen quality: a prospective analysis	Cross-sectional study	409	Marijuana use may have a detrimental effect on semen quality, particularly morphology and volume, but maybe protective against abnormal sperm motility.
Har-Gil et al. [30]	2021	The relationship between cannabis use and IVF outcome—a cohort study	Cohort study	722	The results may be reassuring for the lack of any demonstrable detrimental effects of cannabis consumption on IVF outcomes. Limitation to this study: retrospective nature, self-reporting of cannabis use, and a small user sample size.

## Review

This section discourses the endocannabinoid system and its association with marijuana regarding the male reproductive system and the aspects of fertility that may or may not be impacted due to marijuana. The clinical studies that align with this postulation were included. The objective is to enumerate the effects of cannabis on the male reproductive system in light of the semen parameters, including the sperm morphology, motility, count, volume, and organic causes that may have an association with infertility in men. The limitations of this study are also enumerated.

The endocannabinoid system: a synopsis

The ECS is a cell-signaling multiplex apparatus discovered by researchers studying THC, a well-known cannabinoid, in the early 1990s. This cannabinoid is the key chemical found in cannabis, a.k.a. marijuana [31]. Hitherto we have understood the crucial functions of this complex system that helps in regulating stress response pathways, pain, mood, the sleep-wake cycle, hunger, and other organ systems, including the cardiovascular, gastrointestinal, skeletal muscle, hepatic, urologic systems, and reproduction, and fertility [32]. In order to understand the mechanism and importance of this system better, we need to know there are three principal elements: endocannabinoids, receptors, and enzymes [31].

Endocannabinoids are naturally occurring lipids that mirror the effects of THC. There are four endocannabinoids known to date: arachidonoylglycerol ether, virodhamine, N- arachidonoylethanolamine or anandamide (AEA), and 2-arachidonoylglycerol (2-AG) [22]. The AEA and 2-AG are the well-known endogenous biolipids deemed vital in anthropoids and are released when required by the body as part of our homeostasis [22].

The cannabinoid receptors are found in a dispersed pattern throughout the body, including the central and peripheral nervous systems and non-nervous tissue [31]. There are two subtypes of CB receptors identified: CB1 and CB2 [19]. CB1 receptors are present in the central nervous system (hippocampus, cerebellum, and striatum, and sparsely in the thalamus [33]), the plasma membrane of the acrosomal region/midpiece/tail of spermatozoa [22], ovary, testis, vas deferens, and other peripheral endocrine tissues [19]. In humans, CB2 receptors are typically found in the peripheral nervous system and prostate epithelium [33,34]. Endocannabinoids can connect to either of both receptors, depending on the location, to produce a suitable response [33].

**Figure 5:**
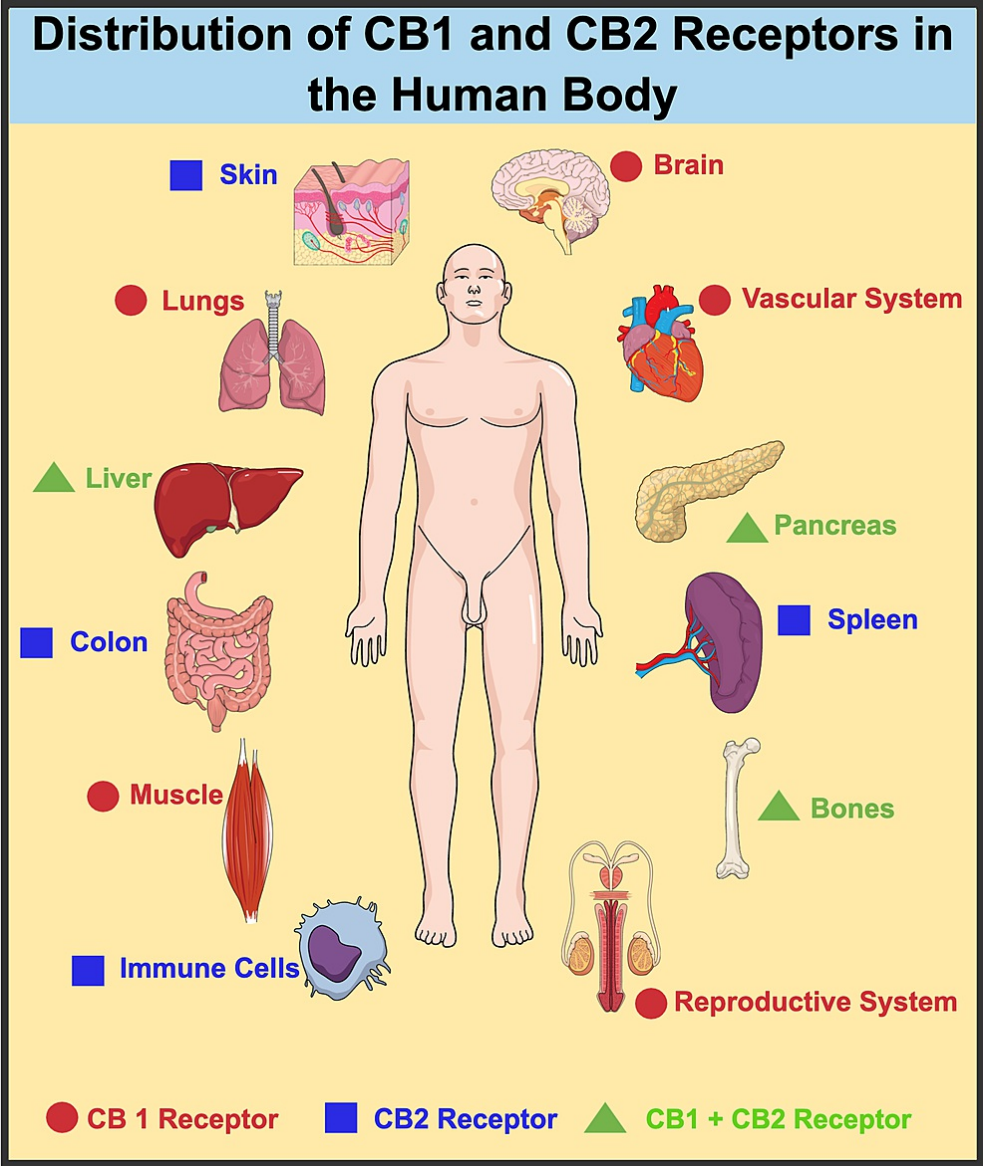
Distribution of CB1 and CB2 receptors in the human body Image created in Mind the Graph platform

Figure 5 depicts the distribution of CB receptors in the human body [35].

Once the endocannabinoids have effectuated their function, they are metabolized by the enzymes. There are two essential enzymes involved in this breakdown process including fatty acid amide hydrolase (FAAH) and monoacylglycerol acid lipase (MGL) [31].

The FAAH is primarily responsible for the degradation of AEA in the CNS and the degradation of multiple fatty acid amides, including palmitoyl and oleoyl ethanolamide [36,37]. Apart from the FAAH, there is an alternative pathway for AEA degradation via oxidation by cyclooxygenase-2 (COX-2) to create prostamides. However, these components are independent of the CB receptors and have been used in other clinical vignettes [37]. The MGL, along with the two other hydrolytic enzymes, alpha/beta-hydrolase domain containing 6 (ABHD6) and 12 (ABHD12), are primarily responsible for the degradation of 2-AG [36]. The COX-2 and FAAH may also aid in the hydrolyzation of 2-AG under certain conditions [37].

How does marijuana act on the ECS?

There are said to be two components that trigger the response of one's body to produce the effects of the ECS, namely, tetrahydrocannabinol and cannabidiol (CBD) [31]. THC is an active ingredient in marijuana. THC is the chemical that causes the "marijuana high", feelings of relaxation and contentment, by binding to the CB1 and CB2 receptors, thus reducing the pain and stimulating appetite. However, in some cases, there may be paranoia and anxiety [31]. CBD is the other key component of cannabis. This chemical does not cause the "marijuana high" or the negative effects. In contrast, it inhibits the degradation of the endocannabinoids and prolongs the action of these endogenous lipids. Some studies conjecture that CBD may interact with receptors that are yet to be discovered [31].

Marijuana and the male reproductive system

The presence of exogenous cannabinoids, such as those present in marijuana, can compete for the CB receptors, which in turn could interfere with the entire ECS and may ultimately result in an imbalance that could eventually impact fertility [38]. Numerous researches conclude that this imbalance exists due to ECS components in the seminal plasma, male reproductive tissues, Leydig and Sertoli cells, and male germ cells [19]. The vicinity of the CB receptors to the hypothalamus exerts a vital role since the hypothalamus is responsible for producing gonadotropin-releasing hormone (GnRH) [21]. The AEA and THC have shown the potential to inhibit gonotrophic release in men through interaction with gamma-aminobutyric acid (GABA), which controls the male reproductive system and function of spermatozoa [22]. In due course, this may bring about a defect in the male reproductive process through various mechanisms.

The summary statistics of individual studies and their association with male fertility are outlined in Table 3.

**Table 3:** Summary of observational study results: relationship of marijuana use with male infertility AEA, anandamide; 2-AG, 2-arachidonoylglycerol; CI, confidence interval; OR, odds ratio; aOR, adjusted OR; FSH, follicle-stimulating hormone

Author	Country/Year	Study method	Level of evidence	Summary statistics	Association with male fertility
Ugboma et al. [18]	Nigeria/2012	Case-control study	IV	The study findings concluded that men who smoke marijuana regularly or have been exposed to it since adolescence showed double the risk of cancer, erectile and infertile abnormalities compared to men who never smoked marijuana.	Associated with erectile dysfunctions
Lewis et al. [19]	Ireland/2012	Cohort study	IV	The study findings showed a marked reduction in AEA and 2-AG content in infertile seminal plasma, along with increased degradation: biosynthesis ratios of both substances in sperm from infertile versus fertile men.	An association exists, as the reduction in endocannabinoid levels in infertile seminal plasma is equivalent to increased degradation.
Pacey et al. [20]	United Kingdom/2014	Case-control Study	IV	After adjustment for other risk factors, men aged ≤30 years using cannabis within the last three months prior to sample collection showed poor sperm morphology (OR = 1.94, 95% CI 1.05–3.60). Men who produced a sample after six days of abstinence were less likely to show poor sperm morphology (OR = 0.64, 95% CI 0.43–0.95).	Abnormal morphology
Gundersen et al. [23]	Denmark/2015	Cross-sectional study	VI	After adjusting for covariates, regular marijuana use more than once a week was linked to a 28% (95% CI −48, −1) lower sperm concentration and 29% (95% CI −46, −1) lower total sperm count. The combined use of marijuana and other recreational substances more than once per week lowered sperm concentration by 52% (95% CI −68, −27) and total sperm count by 55% (95% CI −71, −31).	Decreased concentration and total sperm count
Thistle et al. [24]	United States/2017	Cross-sectional study	VI	No difference was found in serum testosterone between ever users (adjusted mean = 3.69 ng/mL, 95% CI 3.46–3.93) and never users (adjusted mean = 3.70 ng/mL, 95% CI 3.45–3.98) on multivariable analysis.	No relationship with fertility outcomes; however, increased serum testosterone was found.
Nassan et al. [26].	United States/2019	Longitudinal study	IV	After adjustments to potential confounders, it was found that sperm concentration was significantly higher (62.7 million/mL) in men who had ever smoked marijuana (N = 365) than men who had never smoked marijuana (N = 297; 45.4 million/mL). However, no significant differences were found in sperm concentration between current (N = 74; 59.5 million/mL) and past marijuana smokers (N = 291; 63.5 million/mL; p = 0.60). Marijuana smokers had significantly lower FSH concentrations than never marijuana smokers (−16% [−27%, −4%]), with no significant differences between current and past marijuana smokers (p = 0.53).	No significant association with sperm concentration; low FSH in marijuana smokers
Carroll et al. [13]	Jamaica/2019	Cross-sectional study	VI	Those with recent marijuana use were 2.6 times (aOR = 2.6; 95% CI 1.0–6.8, p = 0.044) and consumers of substantial amounts of marijuana were 4.3 times (aOR = 4.3; 95% CI 1.1–15.9, p = 0.030) more likely to be diagnosed with abnormal motility (asthenozoospermia). Furthermore, men who consumed marijuana in moderation were 3.4 times (aOR = 3.4; 95% CI 1.5–7.9, p = 0.004) more likely to be diagnosed with abnormal morphology (teratozoospermia).	Decreased sperm motility and abnormal morphology
Hehemann et al. [29]	United States/2021	Cross-sectional study	VI	The multivariate logistic regression analyses showed current use was associated with increased odds of abnormal morphology (OR 2.15, 95% CI 1.21–3.79], reduced semen volume (OR 2.76, 95% CI 1.19–6.42), and reduced sperm motility (OR 0.47, 95% CI 0.25–0.91), where the latter two were lesser than the WHO reference value.	Abnormal morphology and reduced semen volume but may be protective against abnormal sperm motility
Har-Gil et al. [30]	Canada/2021	Cohort study	IV	The study results showed a similar implantation rate (40.74% vs. 41.13%) and ongoing pregnancy rate (35.2% vs. 29.1%) between users and non-users.	No significant outcome

Sperm count and sperm concentration

A cross-sectional study conducted in Denmark with the target population undergoing fitness examination for military service showed 28% lower sperm concentration and a 29% lower sperm count when comparing men using marijuana more than once per week to men who had never used marijuana [23]. Kolodny et al. studied 20 men (18-28 years of age) who smoked marijuana four days per week for six months. This study resulted in a significantly lower average sperm count in men who used marijuana 10 or more times a week than men who smoked only five to nine times a week [39]. This shows a dose-related pattern that could affect the sperm count. The time taken for sperm count to decrease was demonstrated by another study where 16 chronic marijuana smokers, exposed to high-dose substances with 8-20 cigarettes per day, showed reduced sperm count in five to six weeks after initiating marijuana [40].

Sperm morphology

The Men's Health Center, located at the University of Washington, conducted a study for infertility evaluation that exhibited a twofold increased risk of strict aberrant morphology [29]. In comparison to the general population, the prevalence of marijuana usage was 43%, with previous use being more prevalent than the current use. Previous users had the highest chances of strict morphology deficits, indicating a delayed detrimental effect of marijuana on sperm morphology [29].

The study conducted by Pacey et al. at a fertility clinic in the United Kingdom during 2014 showed abnormal morphology where <4% constituted normal forms in younger men who were using marijuana for three months prior to sample collection. However, men who abstained from use for at least six days were less likely to show abnormal morphology [20]. A study conducted in 1978 by Zimmerman et al. suggested that cannabis use could affect sperm morphology and displayed irregular shapes of heads when tested on mice with an intraperitoneal injection of marijuana for five consecutive days [16]. An animal study in 2010 established a relationship between the cannabinoid receptors in mouse spermatids to influence the chromatin remodeling, thus affecting the morphology [41]. Even though not tested in humans, this postulation could be a possible mechanism of teratozoospermia (abnormal morphology) in cannabis users.

A longitudinal study conducted in the United States in 2019 by Nassan et al. set forth a contradictory result on the link between marijuana use and sperm quality, DNA fragmentation, and serum reproductive hormones [26]. The evaluation of 662 subfertile men showed no clinical significance between ever marijuana users and never marijuana users. However, a statistical significance was established; there was no difference in sperm count, concentration, morphology, and motility on a par with the reference set by WHO for the above-mentioned parameters [26].

Sperm motility

Due to the downstream signaling through the CB1 receptor [22], the sperm motility was diminished and could have an inhibitory effect especially found in recent and high-dose consumers of marijuana [13]. An in vitro study performed in 2006 demonstrated a decrease in sperm motility in response to doses of the delta-9-THC in recreational levels, which again supports the effect of diminished motility [42].

Studies have emerged to show that CB2 can regulate spermatozoa's motility [43]. The activation of this receptor could impede the sperm and cause a sluggish progression while CB1 activity increases the immobile sperm [22]. Since humans possess endogenous agonists, both CB1 and CB2 receptors are activated and reflect a dose-dependent motility response. Exogenous cannabis is significant in this process as it may induce an unintended reduction in spermatozoa motility [43]. In turn, poor motility could result in improper capacitation in the female reproductive tract before meeting the oocyte [43]. Amoako et al. studied 86 men and concluded that AEA reduced sperm motility and viability, which could be most likely due to CB1-mediated suppression of mitochondrial function [38].

In one study, men who ever used marijuana showed improved odds of normal sperm motility surpassing the WHO reference value than men who never consumed marijuana [29]. The study contradicts the theory that cannabis affects semen parameters concerning motility alone since the other sperm parameters were detrimental.

Sperm capacitation

In order to penetrate and fertilize an oocyte, the sperm undergoes a physiological process known as capacitation [44]. In one of the studies, it is premised that AEA plays a significant role in controlling sperm capacitation. The process of capacitation occurs due to an increase in sperm calcium concentration via the transient receptor potential vanilloid 1 (TRPV1) channels [19]. The TRPV1 channels are expressed in the human spermatozoa, which serves as a vehicle for sperm thermotaxis [45]. However, in an infertile sperm, the TRVP1 channel activity is absent, along with reduced levels of AEA in the seminal plasma, which mitigates AEA's fertilizing capability [19]. The average sperm with its fully functioning ECS maintains an uncapacitated form until the sperm meets the oocyte. The mimicking action of THC to the endocannabinoids impairs the CB1 signaling pathway, in turn leading to premature capacitation in freshly ejaculated sperm that could also result in infertility due to the lack of proper penetration of the oocyte by the sperm. Furthermore, a reduction in TRPV1 may moderately account for oligospermia in infertile men [19].

Testosterone

Studies conducted in the 1970s-1980s did not show any conclusive association between cannabis and serum testosterone, and the positive association mainly relied on animal studies [16]. It was not until the recent decade that studies started to show some association between these two variables.

A study conducted in Nigeria in year 2012 evaluated frequent and long-time users of marijuana visiting the urology clinics. The use resulted in decreased levels of testosterone, luteinizing hormone (LH), follicle-stimulating hormone (FSH), as well as decreased testicular size. Some chronic users even reported gynecomastia [18]. This shows that marijuana has an excellent affinity for reproductive organs and could bring about disorders such as erectile dysfunction (ED) and nonseminoma testicular cancers, especially if marijuana was used since adolescence [18].

The testosterone level was also decreased in another study where marijuana was more frequently used and it was established as a dose-dependent reduction [39]. Albeit, an increase in testosterone level back to normal was seen in individuals who were abstinent [39]. The inverse relation of testosterone and cannabis use is also established in the study by Thistle et al., where the recent use of marijuana showed decreased testosterone levels compared to frequent use [24]. On the other hand, a cross-sectional study showed increased serum testosterone levels by 7% compared to marijuana users and non-users, with decreased overall sperm quality [23].

Follicular stimulating hormone

The study by Ugboma et al. supports the reduction in FSH levels. A 16% lower serum FSH concentration was seen in men who had ever smoked marijuana than men who had never smoked, with no significant differences between past and current users [18]. Plasma FSH was also found to be lower than normal in the study by Kolodny et al., where the intervention group consisted of men who smoked five to nine marijuana cigarettes per week and another group who smoked 10 or more marijuana cigarettes per week, with the study showing a statistical significance for the group who smoked 10 or more marijuana cigarettes per week (p<0.01) [39].

Other studies, such as those by Cone et al. and Vescovi et al., did not find a significant reduction in FSH levels with recent use and infrequent chronic use despite intravenously administering GnRH to the latter study group [46,47]. Keep in mind that the sample size, however, was limited in both studies.

Luteinizing hormone

LH levels were significantly lower in the intervention group when compared to the control group in human [46,47] and animal studies [48]. Kolodny et al. were the only researchers to find otherwise normal LH levels in both groups, with one group smoking five to nine cigarettes per week and the other group smoking 10 or more cigarettes per week [39]. It is only fair to say that detailed studies are required to draw a rational conclusion to this finding.

Decreased testicular size

Few animal studies have shown testicular atrophy when exposed to cannabis. It is theorized that this could be due to the oxidative stress caused by the substance, resulting in damage to the seminiferous tubules [16]. Human studies have not yet established this finding except for one study in Nigeria in 2012. The intervention group (weekly marijuana smoker and/or marijuana smokers since adolescence) showed diminished testis size as per the ultrasound scrotal findings [18]. The study, however, was a case-control study with a small number, and further validation would be required to elaborate on this finding.

A note on sexual function

Some studies have shown an increase in the sexual desire when using marijuana due to the activation of the nucleus accumbens, which in turn stimulates the sexual feelings; however, this increase in libido appears to be short term evident from the animal and human studies conducted over the recent years [16]. Aversa et al. concluded in their research that 78% of men who had organic ED admitted to frequently smoking cannabis in contrast to only 3% suffering from non-organic ED [49]. This trend was observed in 64 men who were evaluated for ED [49].

Limitations

Due to ethical considerations, this report mainly focused on observational studies and did not include any randomized controlled trials. All observational studies were of small groups from a multicentric society where the legal status of marijuana may vary. However, more large-scale human trials would be required to reach a definite conclusion on the association of marijuana with male fertility disorders. The studies were restricted to humans only, and no animal studies were reviewed for uniformity. Other urological complications due to marijuana and its active metabolites are yet to be studied in depth to determine a clear-cut association.

## Conclusions

Current human and animal studies declare substantial evidence on impaired male reproductive system on the grounds of sperm parameters, where count/concentration, motility, morphology, capacitation, and viability are affected negatively on marijuana consumption. This conclusion is stressed mainly with regard to men in the subfertile category and men evaluated for infertility disorders. It is also worth noting that hormones may not be affected in all cases of marijuana users. The effect may depend upon the frequency, dose, route, and chronicity of the use. However, no validation is present as all the studies have looked into a small cohort, or are case-control or cross-sectional studies, which may incorporate numerous biases like recall and self-reporting biases. In order to ascertain a tangible sequela, further studies are required, preferably clinical trials in a larger population, taking into account the ethical concern and affirming the generalizability of the trials. Meanwhile, it is recommended that all clinicians beware of these potential effects while evaluating men or couples with infertility disorders and navigate a path where the usage of marijuana could be terminated when and where possible while also keeping in mind the possible adverse effects that could arise before prescribing cannabis to a patient of this concern.
